# Influence of resting lung diffusion on exercise capacity in patients with COPD

**DOI:** 10.1186/s12890-017-0454-y

**Published:** 2017-08-25

**Authors:** Mehrdad Behnia, Courtney Wheatley, Alberto Avolio, Bruce Johnson

**Affiliations:** 1 University of Central Florida School of Medicine and Division of Critical Care, Florida Hospital, Orlando, FL USA; 2 Division of Cardiovascular Diseases, Mayo Clinic, Scottsdale, AZ USA; 3 Faculty of Medicine and Health Sciences, Macquarie University, Sydney, NSW Australia; 4 PO Box 953814, Lake Mary, FL 32795 USA

**Keywords:** Lung surface area, Gas exchange, Dyspnea, Gas transfer

## Abstract

**Background:**

Lung diffusing capacity for carbon monoxide (DLCO) gives an overall assessment of functional lung surface area for gas exchange and can be assessed using various methods. DLCO is an important factor in exercise intolerance in patients with chronic obstructive pulmonary disease (COPD). We investigated if the intra-breath (IBDLCO) method may give a more sensitive measure of available gas exchange surface area than the more typical single breath (SBDLCO) method and if COPD subjects with the largest resting DLCO relative to pulmonary blood flow (Qc) would have a more preserved exercise capacity.

**Methods:**

Informed consent, hemoglobin, spirometry, SBDLCO, IBDLCO, and Qc during IBDLCO were performed in moderate to severe COPD patients, followed by progressive cycle ergometry to exhaustion with measures of oxygen saturation (SaO_2_) and expired gases.

**Results:**

Thirty two subjects (47% female, age 66 ± 9 yrs., BMI 30.4 ± 6.3 kg/m^2^, smoking hx 35 ± 29 pkyrs, 2.3 ± 0.8 on the 0-4 GOLD classification scale) participated. The majority used multiple inhaled medications and 20% were on oral steroids. Averages were: FEV_1_/FVC 58 ± 10%Pred, peak VO_2_ 11.4 ± 3.1 ml/kg/min, and IBDLCO 72% of the SBDLCO (*r* = 0.88, SB vs IB methods). Using univariate regression, both the SB and IBDLCO (% predicted but not absolute) were predictive of VO_2_peak in ml/kg/min; SBDLCO/Qc (*r* = 0.63, *p* < 0.001) was the best predictor of VO_2_peak; maximal expiratory flows over the mid to lower lung volumes were the most significantly predictive spirometric measure (*r* = 0.49, *p* < 0.01). However, in multivariate models only BMI added additional predictive value to the SBDLCO/Qc for predicting aerobic capacity (*r* = 0.73). Adjusting for current smoking status and gender did not significantly change the primary results.

**Conclusion:**

In patients with moderate to severe COPD, preservation of lung gas exchange surface area as assessed using the resting SBDLCO/Qc appears to be a better predictor of exercise capacity than more classic measures of lung mechanics.

## Background

Causes contributing to exercise limitation in patients with chronic obstructive pulmonary disease (COPD) are complex [1–3]. Previous studies have suggested that while lung mechanics clearly play an important role, there are many other factors that contribute to this limitation such as heterogeneity of the disease process, lifestyle issues, such as weight and activity patterns, deconditioning, disease-related inflammatory processes, perception, as well as associated comorbidities such as cardiovascular disease [4–6]. Pulmonary function measures representing the degree of obstruction and severity of hyperinflation (e.g., inspiratory capacity or IC) appear important as well as less appreciated factors such as a blunted cardiac output, either due to airway obstruction and rise in intra-thoracic pressure or from the development of pulmonary hypertension [7–9]. As a result, exercise capacity as a whole has been used as a prognostic indicator in the COPD population and as such is a good assessment of the integrative factors involved in the disease [10]. In addition, as stated by the GOLD initiative (Global Initiative for Chronic Obstructive Lung Disease classification for air flow obstruction), improvement in exercise tolerance is recognized as an important goal of COPD treatment.

From the lung volume reduction surgery data, it has also been found that certain patterns of disease and perhaps more severe emphysema may be associated with worse exercise tolerance [11, 12]. Of the common relatively simple screening tests, a low lung diffusing capacity for carbon monoxide (DLCO) has been shown to not only suggest a more emphysematous pathophysiology but has also been a predictor of exercise capacity and in particular exercise induced oxygen desaturation [1, 13]. There are different ways to quantify DLCO, from the typical single breath method (SBDLCO), to various rebreathe, steady state, open-circuit and intra-breath techniques [14]. While the latter methods may represent in some sense more physiological quantification of functional lung surface area for gas transfer or exchange, the single breath method has been standardized with well-established predictive norms for clinical use [15].

The intra-breath method (IBDLCO) is interesting in that it potentially represents a relatively simple way to quantify DLCO in patients that may struggle with longer breath hold times and there is potential for use during exercise to quantify alveolar–capillary surface area recruitment. It requires the exhalation of test gas typically near residual volume, followed by a deep inhalation and essentially instantaneous exhalation back towards residual volume (RV). The expiratory sampling relies on a fast response CO analyzer and as exhalation continues towards RV, the DLCO at any point is dependent then on the exhaled lung volume and each time point would represent a DLCO that is a mix of CO uptake and mixing with other lung gases [16, 17]. An advantage of this technique compared to other more discrete techniques is that the exhaled gas stream is used in its entirety to calculate DLCO.

In patients with lung disease it is likely that the intra-breath method may be more sensitive to disease pathology relative to the single breath method due to abnormalities in ventilation and perfusion and delayed time constants for ventilation with the shortened gas exchange times.

Lung diffusion is dependent on pulmonary capillary blood volume (Vc) and alveolar-capillary gas exchange surface area, usually reported as membrane diffusion capacity. While these components of DLCO can be estimated by performing DLCO at multiple oxygen concentrations or with a second gas such as nitric oxide (DLNO), a surrogate may be obtained for blood volume by examining the DLCO relative to cardiac output (Qc). Since a rise in Q tends to be the major reason for distension or recruitment of capillaries, a larger ratio would be indicative of a healthier phenotype.

Thus in the present study we were interested in the role of resting DLCO in predicting exercise capacity in a relative diverse group of COPD patients. More specifically we were interested if a higher intra-breath to single breath DLCO ratio or a higher DLCO relative to Qc ratio would better predict exercise capacity relative to other common measures of lung mechanics. We hypothesized that those subjects with a higher IBDLCO relative to SBDLCO or a higher SBDLCO/Qc ratio would have better preserved exercise capacity.

## Methods

### Ethics and consent

The study, ethics, and consent forms were reviewed and approved by the Western Institutional Review Board (WIRB, study number 1153374).

### Subjects

Patients with a history of COPD that were sent for clinical pulmonary function testing and/or exercise testing were offered enrollment. Inclusion criteria included established patients with a history of COPD, on stable medications without recent exacerbation (within 3 months). Exclusion criteria included, oxygen dependence an inability to exercise and/or a BMI > 42. Both past and current smokers were allowed to participate with 7 of the participants being current smokers. Prior to participation, the study goals and requirements were reviewed with the patients. If willing to participate, patients signed informed consent.

### Overview of study

After reporting to the outpatient clinic, study participants filled out the St. George’s Respiratory quality of life questionnaire (SGQOL), performed pulmonary function testing (PFTs) which included resting measures of maximal lung volumes and flow rates using classical spirometry. In addition the assessment of lung diffusing capacity for carbon monoxide (DLCO) was obtained using the classical single breath (SB) technique and was also obtained using the intra-breath method (IB) which included a measure of pulmonary blood flow (Qc). A small blood sample was obtained prior to testing for assessment of hemoglobin in order to correct the measure of DLCO. Subjects subsequently performed cardiopulmonary exercise testing (CPET) using the CareFusion Vmax Encore metabolic cart (San Diego, CA) with a Corival recumbent cycle ergometer (Lode, Netherlands). The test protocol started with 20 watts for both men and women and increased by 10 watts every 2 min. Prior to exercise testing, subjects were instrumented with a 12 lead ECG, and a forehead pulse oximeter for peripheral oxygen saturation (SaO_2_) for continuous monitoring. Subjects wore a nose clip and breathed on a mouthpiece for continuous measurement of gas exchange during the exercise test. During the last 30 s of each workload, a 12 lead ECG recording was printed, blood pressure (BP) assessed, perceived dyspnea score (0-10 scale) and perceived exertion (an assessment of total body effort) was rated by subjects, and an average of the HR and SpO_2_ over this period was determined. The goal was to obtain at least 2–3 work levels for each subject. Subjects were encouraged to exercise to near exhaustion based on symptom limitation by achieving an RPE of 17-18 on the Borg 6-20 scale or a dyspnea score ≥ 7 on the 0-10 score [18]. Upon reaching peak symptom limited exercise, subjects performed active recovery where they continued to pedal with no resistance and remained on the mouthpiece for 1 min. After this the subject stopped pedaling and was given time for HR and BP to return to baseline before being dismissed.

### Pulmonary function and single breath DLCO

Spirometry was performed using pneumotachograph-based pulmonary function equipment that has passed evaluation using 24-wavefroms recommended by the American Thoracic Society (ATS). Classic single breath DLCO was determined using a commercial instrument that utilizes a gas chromatograph to analyze expired gas samples, following the recommendations of the ATS/ERS [15, 19].

### Intrabreath lung diffusing capacity and pulmonary blood flow (qc)

Pulmonary Blood Flow (Qc) and diffusing capacity of the lungs for carbon monoxide (DLCO) were measured using inert and soluble gases on the CareFusion Vmax system using an intra-breath maneuver [17]. For this maneuver, subjects were asked to breath on a mouthpiece while wearing a nose clip. Subjects were instructed to exhale to residual volume (RV) and then were switched in to an inspiratory reservoir and took a maximal inhalation of a test gas mixture containing 0.3% carbon monoxide (CO), 0.3% methane, 0.3% acetylene, 21% O_2_, and balance N_2_. Subjects were coached to exhale slowly at a steady rate until they were near RV. From the rate of disappearance of CO and acetylene in comparison to the inert gas methane the rate of disappearance of CO and acetylene were determined. This rate of disappearance of CO provides the DLCO value. Since acetylene does not bind to hemoglobin the rate of its disappearance is limited only by the flow of blood through the lungs, thereby providing a measure of Q [20, 21].

### QOL questionnaires. St. George’s respiratory questionnaire

The SGRQ is a 50-item questionnaire developed to measure health status (quality of life, QOL) in patients with diseases of airways obstruction. Scores are calculated for three domains: Symptoms, Activity and Impacts (Psycho-social) as well as a total score. Psychometric testing has demonstrated its repeatability, reliability and validity. Sensitivity has been demonstrated in clinical trials. A minimum change in score of 4 units has previously been established as clinically relevant. The SGRQ has been used in a range of disease groups including asthma, chronic obstructive pulmonary disease (COPD) and bronchiectasis, and in a range of settings such as randomized controlled therapy trials and population surveys [22]. The SGRQ correlates significantly with other measures of disease activity such as cough, dyspnea, 6-min walk test and FEV_1_ as well as other measures of general health such as the Sickness Impact Profile (SIP) score which evaluates the impact of disease on physical and emotional functioning and Short Form 36 (SF36) health survey which is a patient reported survey of health [23].

### Gas exchange, ventilation and lung mechanics

During exercise testing oxygen consumption (VO_2_), carbon dioxide production (VCO_2_), breathing frequency (fb), tidal volume (V_t_), minute ventilation (V_E_) and derived variables (e.g., V_E_/VCO_2_) were measured continuously or calculated using a low resistance open circuit automated metabolic system (CareFusion).

#### Statistics

We were interested in the association of resting measures of DLCO measured via single breath or intra-breath methods as well as expressed relative to Qc with exercise capacity (peak VO_2_) in patients with moderate to severe COPD and if these measures were more highly associated to exercise capacity than more typical measures of lung mechanics. Descriptive statistics were used to describe patient characteristics and demographics while multiple regression and correlational analysis were used to determine associations between DLCO, Q, lung mechanics, QOL, disease severity and exercise capacity. Statistics were performed with a combination of EXCEL and the statistical software package JMP Statistical Discovery TM software from SAS.

## Results

### Subject characteristics and pulmonary function measures

Thirty two subjects completed the study. As shown in Table 1, on average our study cohort was older, approximately half female, above ideal body mass index and had a 35 pack year smoking history. By design, their GOLD classification ranged from 1 to 4 with an average classification consistent with moderate disease with an FEV_1_ of 56% of age predicted and an FEV_1_/FVC ratio of 59% (Table 2). None of the subjects were on continuous oxygen or oxygen for exercise at the time of the study. The majority of subjects were on combination inhalation therapy that included inhaled beta-2 agonist, anticholinergic, and inhaled steroid with a minority of subjects on oral steroids. Quality of life scores based from the St George questionnaire was consistent with severity of disease as described by the GOLD classification.

**Table 1:** Subject characteristics (*n* = 32)

	Mean ± SD	Range
Age (years)	66 ± 9	46 - 84
% Female	47	-
Weight (Kg)	88 ± 23	36 - 155
BMI (Kg/m2)	30 ± 6	13 - 44
Smoking history (pack year)	35 ± 29	0 - 120
Current/former/never smoker (n)	6/22/4	-
GOLD Classification (1–4)	2.3 ± 0.8	1 - 4
St George Respiratory Questionnaire	44 ± 21	8 - 84
Inhaled beta agonist (%)	97	-
Inhaled anticholinergic (%)	59	-
Inhaled steroid (%)	68	-
Oral steroid (%)	20	-

*GOLD* Global Initiative for Chronic Obstructive Lung Disease classification for air flow obstruction

**Table 2:** Pulmonary Function Variables

	Mean ± SD	Percent Predicted (range)
FVC (L)	2.48 ± 0.69	75 ± 15
FEV_1_ (L)	1.51 ± 0.58	56 ± 16
FEV_1_/FVC	59 ± 11	(33 – 78)
FEF _25-75_ (L/s)	0.75 ± 0.38	26 ± 13
FEF_75_ (L/s)	0.29 ± 0.11	27 ± 13
MVV (L/m)	48 ± 19	45 ± 17

*FVC* Forced Vital Capacity, *FEV*_*1*_ Forced Expiratory Volume in 1 s, *FEF* Forced Expiratory Flow, *MVV* Maximal voluntary ventilation. All data are pre bronchodilator

### Resting lung diffusion measures – Single breath vs intra-breath

**Fig. 1:**
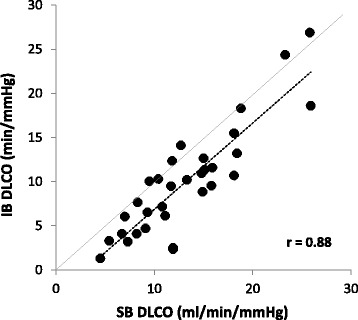
Relationship of Single Breath DLCO (SBDLCO) to Intra Breath DLCO (IBDLCO, *n* = 32). The IBDLCO was on average lower than the SBDLCO (*p* < 0.001) particularly in patients with values that were more significantly reduced relative to predictive values (<65% of predicted)

**Fig. 2:**
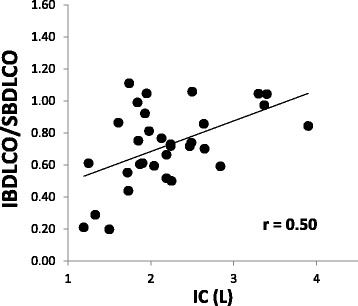
Relationship of inspiratory capacity (IC) to the IBDLCO and SBDLCO ratio in patients with COPD (*n* = 32). Subjects with the highest IC tended to have the highest ratio of IB to SBDLCO

Table 3 lists single breath and intra breath DLCO measures, the measured pulmonary blood flow (Qc), and Hgb values. SBDLCO averaged 13.2 ml/min/mmHg and 58% of predicted with the average IBDLCO 71% of the SB method ranging from 20 to 110% across the study population. Overall the SB and IB methods were highly correlated with an r of 0.88 (Fig. 1) and the IB/SBDLCO relationship was positively associated with resting IC (Fig. 2). The measured pulmonary blood flow (Qc) using soluble gas was 76% of the resting predicted cardiac output based on gender and body size. On average the SBDLCO was 51% predicted in current smokers vs 56% predicted in those that had quit or never smoked. Though the current smokers were slightly reduced relative to nonsmokers, there was no statistical difference between groups (*p* < 0.05).

**Table 3:** Lung diffusing capacity and pulmonary blood flow

	Mean ± SD	Percent predicted or (Range)
Single Breath DLCO (SBDLCO, ml/min/mmHg)	13.2 ± 5.5	58 ± 23 (31 –112)
Intra Breath DLCO (IBDLCO, ml/min/mmHg)	9.7 ± 5.9	(1.3 – 27)
IBDLCO/SBDLCO (%)	71 ± 26	(20 – 110)
Pulmonary Blood Flow (Qc, L/m, measured)	4.8 ± 0.9	(3.3 – 6.8)
Pulmonary Blood Flow –Cardiac output, (L/m, Predicted)	6.3 ± 0.4	(5.4 – 7.1)
SBDLCO/Qc ratio	2.8 ± 1.2	(1.2 – 5.7)
Hgb (g/dl)	13.5 ± 1.7	(11–17)

Pulmonary Blood Flow measured with soluble gas method. Cardiac output estimated based on age, gender, BSA, from Ref (William LR). Qc = Pulmonary Blood Flow

### Cardiopulmonary exercise responses

Cardiopulmonary exercise responses are reported in Table 4. On average the peak VO_2_ for the group was 0.98 L/min or 11.4 ml/kg/min equivalent to 50% of age and gender predicted. Average peak heart rate was 103 bpm which was 70% of their age predicted and average respiratory exchange ratio (RER) was 1.03. Inspiratory capacity consistently fell throughout exercise and at peak the tidal volume reached 65% of the IC. Oxygen pulse rose to an average of 8 ml/beat early in exercise but then plateaued thereafter and reached a max of 9.1 in peak exercise suggesting a plateauing of cardiac stroke volume. Subjects complained of both general fatigue and dyspnea as major reasons for stopping the test. At peak exercise the minute ventilation averaged 73% of the maximum voluntary ventilation (MVV) with five subjects exceeding their pre-test MVV.

**Table 4:** Breathing pattern, lung mechanics and gas exchange responses to exercise (*n* = 32)

	Rest	First work load	Peak exercise
Heart Rate (bpm)	77 ± 10	92 ± 11	103 ± 22
RPE (6–20)	7 ± 2	11 ± 2	17 ± 2
Dyspnea (0-10)	1 ± 1	3 ± 2	7 ± 2
VE (L/min)	12.5 ± 2.7	24 ± 6	34 ± 11
Fb/VT ratio	26 ± 15	26 ± 14	30 ± 13
TI/TTOT ratio	39 ± 8	38 ± 4	39 ± 4
IC (L)	2.2 ± 0.7	1.9 ± 0.7	1.8 ± 0.7
VT/IC (%)	37 ± 13	52 ± 12	65 ± 27
VO_2_ ml/kg/min	3.7 ± 0.8	8.3 ± 1.4	11.4 ± 3.1
VE/VCO_2_ ratio	47 ± 7	37 ± 5	36 ± 5
PetCO_2_ mmHg	35 ± 5	37 ± 4	37 ± 5
O_2_Pulse	4 ± 1	8 ± 2	9 ± 3
SaO_2_ (%)	96 ± 2	95 ± 3	94 ± 3

*VE* Minute ventilation, *fb* breathing frequency, *VT* tidal volume, *TI* inspiratory time, *TTOT* total respiratory cycle time, *IC* inspiratory capacity, *VO*_*2*_ oxygen consumption, *VCO*_*2*_ carbon dioxide production, *PetCO*_*2*_ end tidal partial pressure of carbon dioxide, *O*_*2*_ *pulse* VO_2_/heart rate, *SaO*_*2*_ arterial oxygen saturation estimated from pulse oximetry

### Relationship of resting measures of lung mechanics and lung diffusion to exercise capacity

**Fig. 3:**
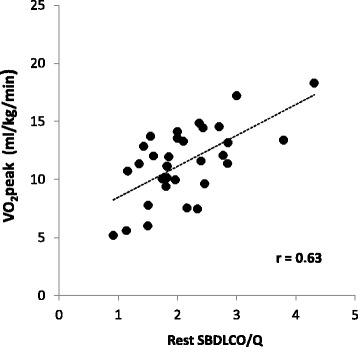
Relationship of SBDLCO/Qc and Exercise Capacity based on VO_2_peak in patients with COPD. The ratio of lung diffusing capacity to pulmonary blood flow was the best predictor of VO_2_peak in this population

**Fig. 4:**
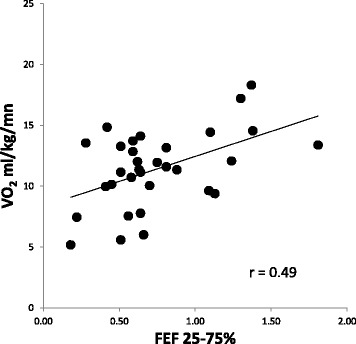
Relationship of FEF _25-75%_ to VO_2_peak in patients with COPD. FEF _25-75%_ was the best univariate lung mechanics predictor of exercise capacity but did not remain in a predictive model when DLCO and weight were added

Univariate correlations of resting measures of lung function, QOL, anthropometric measures and lung diffusion relative to exercise capacity (expressed in ml/kg/min as well as in L/min) are shown in Table 5. The values that were most significantly linked to exercise capacity based on VO_2_peak in ml/kg/min were SBDLCO relative to pulmonary blood flow (SBDLCO/Qc) (Fig. 3), SBDLCO (% Pred), VT/IC, (where VT is tidal volume), absolute measures of FEF_25-75_ (Fig. 4), FEF_75_ and BMI (*p* < 0.01). Also associated but less significantly so (*p* < 0.05 but >0.01) were the IBDLCO also relative to Qc as well as FVC and FEF50%. In a step wise fashion or when allowing all significant variables to compete in a multiple regression, only SBDLCO/Qc and BMI remained in a model predicting VO_2_ peak ml/kg/min where:

$$ \left[{\mathit{\mathsf{VO}}}_{\mathit{\mathsf{2}}}\mathit{\mathsf{peak}}\ \mathit{\mathsf{ml}}/\mathit{\mathsf{kg}}/\mathit{\mathsf{\min}}=\mathit{\mathsf{2}.\mathsf{51}}\ \left(\mathit{\mathsf{DLCO}}/\mathit{\mathsf{Qc}}\right)\hbox{-} \mathit{\mathsf{0.176}}\ \left(\mathit{\mathsf{BMI}}\right)+\mathit{\mathsf{11.48}},\mathit{\mathsf{withan}}\ \mathit{\mathsf{r}}\ \mathit{\mathsf{of}}\ \mathit{\mathsf{0.73}}\ \mathit{\mathsf{an}\mathsf{d}}\ \mathit{\mathsf{an}}\ {\mathit{\mathsf{R}}}^{\mathit{\mathsf{2}}}\mathit{\mathsf{of}}\ \mathit{\mathsf{0.53}}.\right] $$

**Table 5:** Univariate Correlations with VO_2_peak expressed in L/min and ml/kg/min

*N* = 32	VO_2_peak ml/kg/min	*p*-value	VO_2_peak L/min	*p*-value
SB DLCO	0.18	0.312	0.51	0.002
SB DLCO (%Pred)	***0.49***	***0.004***	0.48	0.006
IB DLCO	0.20	0.277	0.51	0.002
IB DLCO (% Pred)	0.41	0.020	0.49	0.004
IB/SB	0.01	0.974	0.37	0.039
**SB DLCO/Qc**	***0.63***	***0.000***	***0.59***	***0.000***
IC (L)	0.40	0.020	***0.60***	***0.000***
VT/IC	***−0.42***	***0.017***	***−0.55***	***0.001***
FVC	0.41	0.018	0.54	0.001
FEV_1_ (% Pred)	0.38	0.030	0.22	0.217
FEF _25-75_ (L/min)	***0.49***	***0.004***	0.48	0.006
FEF _75_ (L/min)	***0.47***	***0.006***	0.41	0.018
Wt (kg)	−0.30	0.092	0.55	0.001
**BMI**	***−0.42***	***0.017***	0.32	0.078
BSA	0.16	0.370	***0.64***	***0.000***
QOL	−0.23	0.200	−0.05	0.773
GOLD classification	−0.27	0.140	−0.20	0.275

*SB* single breath method, *IB* intra-breath method. Values in columns 2 through 5 that are most significantly linked to exercise capacity based on VO_2_ peak are in bold and italics. In column 1, only the variables that predict VO_2_ peak in ml/kg/min in a multiple regression analysis, are in bold.

When expressing VO_2_ peak as L/min, SBDLCO/Qc, IC, VT/IC and BSA were the most predictive of exercise capacity with FVC being the strongest lung mechanics measure in a multiple regression but which lost significance when BSA was added. Thus, the best model for absolute VO_2_ was:

$$ \left[{\mathit{\mathsf{VO}}}_{\mathit{\mathsf{2}}}\mathit{\mathsf{peak}}\ \mathit{\mathsf{L}}/\mathit{\mathsf{\min}}=\mathit{\mathsf{0.2038}}\ \left(\mathit{\mathsf{DLCO}}/\mathit{\mathsf{Qc}}\right)+\mathit{\mathsf{0.6193}}\ \left(\mathit{\mathsf{BSA}}\right)\hbox{-} \mathit{\mathsf{0.665}},\mathit{\mathsf{withan}}\ \mathit{\mathsf{r}}\ \mathit{\mathsf{of}}\ \mathit{\mathsf{0.81}}\ \mathit{\mathsf{an}\mathsf{d}}\ \mathit{\mathsf{an}}\ {\mathit{\mathsf{R}}}^{\mathit{\mathsf{2}}}\mathit{\mathsf{of}}\ \mathit{\mathsf{0.65}}.\right] $$

### Influence of smoking and gender on predictors of exercise capacity

Since six of our cohort were current smokers and current smoking and the time of abstention from smoking is known to impact DLCO [24, 25], we performed both stepwise and multivariate models accounting for smokers. Under both conditions, SBDLCO/Qc and BMI still remained significant predictors (*p* < 0.01) with the influence of smoking being not significant (*p* = 0.67). Subgroup analysis excluding current smokers was also performed relative to VO_2_ peak ml/kg/min, where DLCO/Q and BMI remained significant predictors (*p* = 0.000 and *p* = 0.009 respectively). Percent predicted SB and IBDLCO were also not significantly different between current and past smokers (*p* > 0.05).

We also accounted for gender in the models, including when expressing VO_2_peak in L/min rather than in ml/kg/min. There was no influence of gender on the relationship between SBDLCO/Qc and VO_2_peak. When expressing VO_2_peak in L/min, SBDLCO/Qc and BSA remained the most significant predictors.

## Discussion

### Primary findings

From our study we conclude that while resting measures of hyperinflation and maximal expiratory flows, particularly over the mid to lower lung volumes were predictive of exercise capacity, lung diffusing capacity alone or expressed relative to resting pulmonary blood flow was the most predictive of exercise capacity. Furthermore, when allowed to compete in a multiple regression model, only the SBDLCO relative to Qc and measure of body weight or habitus were significant predictors and explained approximately 50-60% of the variability in exercise capacity in this population.

### Previous studies looking at exercise intolerance in COPD

Factors contributing to exercise intolerance in patients with COPD are complex. While a number of studies have examined predictors of exercise capacity in the COPD population, the majority of these have focused on measures of lung mechanics and while relationships are found between measures of maximal expiratory flows and volumes, measures of hyperinflation appear to be the most predictive [3, 8, 9, 26, 27]. In this study we evaluated several measures of lung function, quality of life, body weight/habitus and measures of lung diffusing capacity measured differently or expressed relative to lung function and pulmonary blood flow. We found that lung mechanics, particularly flows at the mid to lower lung volumes and the inspiratory capacity relationship, or tidal volume inspiratory capacity relationship, seemed to be the most predictive. However when allowed to compete in a model with measures of lung diffusion and measures of body weight or habitus, the measures of mechanics no longer reached significance. In particular when SBDLCO was expressed relative to the measured Qc with BMI or when predicting VO_2_ in L/min, BSA together in a model the mechanics measures, were no longer contributory.

DLCO is a variable of paramount importance in pulmonary medicine. It represents a complex integration of factors including ventilation distribution, matching of ventilation to perfusion, the resistance at the alveolar-capillary membrane as well as the combination rates with hemoglobin. Since all of the above factors can be affected with more classic patterns of emphysema and COPD with ultimate destruction of the alveolar-capillary bed, preservation of DLCO is an important marker of lung health [13]. For example, a major factor contributing to recruitment or distension of the pulmonary capillary bed is cardiac output or pulmonary blood flow (Qc) [28]. As alveolar-capillary walls are remodeled, destroyed or even stiffened with disease, the DLCO/Qc relationship would be altered. With exercise, ventilation rises and pulmonary blood flow increases resulting in elevation of DLCO. In emphysema and COPD with loss of alveolar volume, an important adaption to maintain gas exchange in the face of increased blood cell transit time is a rise in pulmonary capillary blood volume. Thus preservation of this relationship in this population should be a discernible advantage. It was interesting that the IBDLCO, while highly correlated to the SBDLCO, was not as predictive of exercise capacity as the SB method. Our original rational was that since the IB method was performed more quickly and at a lung volume more specific to tidal breathing, that it may be a more sensitive predictor of functional gas exchange surface area. However, during the rapid inspiratory phase of the SBDLCO method, potentially increasing pulmonary blood volume, and with the inhalation to total lung capacity, increasing alveolar volume, it is likely this gives a better overall representation of functional or possibly recruitable surface area available for use during exercise. Also of note was the fact that the IBDLCO method was more variable across subjects and less reproducible than the SB method.

### Other predictors of exercise capacity in COPD

In what have become classical studies by O’Donnell and colleagues, resting IC, degree of hyperinflation with exercise, and change in IC, have all been highly predictive of exercise capacity [9]. Hyperinflation is associated with expiratory flow limitation, volume constraints and less optimal respiratory muscle performance [3, 12, 26]. Dynamic hyperinflation during exercise contributes to perceived respiratory discomfort. Indirect evidence of the importance of dynamic hyperinflation comes from studies that have demonstrated that alleviation of dyspnea following bronchodilator therapy and lung volume reduction surgery (LVRS) are both explained, in part, by reduced operating lung volumes [29]. Additional studies have suggested that COPD patients enter into a spiral of decline associated with reduced activity, inflammation and skeletal muscle dysfunction [2, 30, 31]. A high work and cost of breathing in the setting of elevated operational lung volumes and in some cases excessive expiratory muscle work and also diaphragmatic fatigue contribute to exercise intolerance [30]. COPD also is associated other co-morbidities such as coronary artery disease, pulmonary vascular disease, pulmonary hypertension and right ventricular failure; all of which impair cardiac output and thus compromise oxygen delivery [31]. While the reality is that these collective contributors to exercise limitation in COPD are all integrated and codependent, our work suggests that maintenance of a functional alveolar-capillary bed is an important determinant of patients ability to exercise and likely to carry on normal daily activities.

Targeting the airways and the inflammatory pathways has been the cornerstone of therapy for COPD and emphysema which are accomplished by classes of beta-2 agonists, anticholinergics, and steroids. Using the same rational, targeting diffusing capacity, i.e. the pulmonary vasculature, by medications has been tried. However, the results have been disappointing. For example, sildenafil, a phosphodiesterase type-5 (PDE-5) inhibitor with vasodilatory properties, commonly used in treatment of pulmonary arterial hypertension (PAH), has been tried in COPD patients who did not have pulmonary hypertension. Interestingly, the drug worsened gas exchange, increased the alveolar-arterial oxygen difference, and did not improve exercise capacity, possibly by causing ventilation-perfusion mismatch, indicating the need for more studies and new medications [26].

### Limitations

There are several limitations relative to this study to consider. First, this was a relatively small study in a somewhat heterogeneous group of primarily moderate to moderately-severe COPD patients who performed cycle ergometry to volitional exhaustion. We essentially recruited consecutive patients with a history of COPD, on stable medications, willing to participate with minimal exclusion criteria. To some extent this was by design so that the study population represents a typical mixed tertiary outpatient population. Lung diffusion has been shown to be predictive of exercise capacity in a number of chronic lung and heart conditions and therefore appears to be a good, more generic marker to consider for the COPD population. Larger studies would however be needed to tease out in which specific COPD populations it may be most predictive. Secondly, we used volitional fatigue as a cessation criteria. This resulted in some subjects with lower than typical RER values or other more traditional measures associated with maximal exercise, e.g., heart rate. Cycle ergometry is known to be associated with more local muscle fatigue and may underestimate true maximal exercise. However, we attempted to use similar stopping criteria for our subjects and the same study staff performed all exercise testing which likely resulted in a more uniform representation of peak exercise capacity across subjects. In addition, since mechanical constraint to breathing occurs, some subjects would be unable to hyperventilate to more typical RER values or get to a true cardiovascular limitation. Thus we feel our data are representative of the typical tertiary center testing laboratory where symptom limitation is typically used for stopping criteria.

We also allowed recruitment of current smokers. While subjects were asked not to smoke within 24 h of testing, we did not specifically assess carboxyhemoglobin levels and therefore could not confirm if they were smoking prior to testing. This could have influenced our DLCO measures, though previously Oglivie et al. felt that the effects of increasing COHb were sufficiently small, so that routine correction of DLCO was not necessary [32]. As a result, DLCO was not until more recently clinically adjusted for increases in COHb [33]. We also did not note a difference in percent predicted DLCO between smokers and non-smokers and did not find that current smoking impacted our predictive models. Finally, we used a soluble gas method for calculation of pulmonary blood flow or in the absence of significant shunt or cardiac output. We acknowledge this method may be somewhat dependent on ventilation and perfusion matching in the lungs and therefore may also underestimate actual values.

## Conclusions

In conclusion, exercise limitation in COPD is affected by alveolar-capillary gas exchange impairment which in turn is attributed to impairment of pulmonary circulation. SBDLCO relative to Qc and body weight are better predictors of exercise performance compared to IBDLCO and other respiratory variables in this population. We proposed that lung diffusing capacity, either alone or relative to pulmonary blood flow, is a good measure of pulmonary vascular health in the COPD population and is also a good measure for assessing mechanisms of exercise intolerance in this population. Medical management targeting the pulmonary circulation may help reduce symptoms and improve exercise tolerance in COPD patients.

## Abbreviations

BMI: Body mass index
COPD: Chronic obstructive pulmonary disease
DLCO: Diffusing capacity for carbon monoxide
IB: Intrabreath
Qc: Pulmonary blood flow
SB: Single breath
SGQOL: St George quality of life
VO_2_: Oxygen consumption
